# Event and Comment

**Published:** 1905-06-15

**Authors:** 


THE
### DENTAL REGISTER.

Vol. LIX. June 15, 1905. No. 6.

### EVENT AND COMMENT.

### Dignifying Our Profession.

The editor of the *Pacific Gazette* in a recent issue says, "It has been and still is too easy to become a dentist. Standards are being advanced it is true, but college fees are too low, and too many insufficiently educated men are allowed admission and graduation from our colleges. If our profession is ever to receive a proper remuneration for its services, it must be impressed with a due appreciation of its own worth, in order that the public shall be impressed with the importance of saving all the teeth as long as it is possible."

There can be no question but that the thoroughly-educated gentleman will render a more valuable service to his patrons as a rule than the man who knows only enough to keep him from committing fatal blunders. And yet it very often happens that the earnest and conscientious man with meager intellectual advantages before entering college will overcome his early deficiencies of education by a concentration of his energies until he becomes a highly-successful and useful practitioner. If this fact was universally or even generally true it would be a very strong argument in favor of selecting dental students, because of their mental attitudes rather than their mental capacities. There is no good reason, other things being equal, why the educated student should not become the stronger and more useful practitioner, and he generally does in the long run. But so many students who could barely pass the entrance requirements have turned out to be great students and practitioners, that we rather expect this to happen much oftener than it does. But one of the statements made in the quotation above is that, by inference at least, students would be more appreciative of their abilities if the cost in money of securing them was increased. Here again we meet the same problem, We naturally value that which cost us money or effort. But cost to one person is only relative and not actual. It can't be measured in dollars. One dollar cost one man double as much as another. Some students have all or more money than they need, while others must deny themselves continually in order that they may be able to secure similar advantages. It would be strange indeed should not the student who earns his way through college prize his diploma more than one who does not know the first principles of self-sacrifice. Given two students of equal mental capacity, and the one who makes the greater sacrifice for his professional training is almost certain to carry this spirit of self-sacrifice into the practice of his profession and it would be strange indeed if he did not have a larger ideal of duty and service. Increasing college expenses may bring us better educated students because they must come from homes that can afford to pay the costs of thorough preliminary education, and because such students have been brought up to live well at home they will want to live well professionally and may value their services to the extent of requiring larger fees for services rendered. But that they will possess a greater reverence for or appreciation of the value of the dental structures with which they must spend their lives than their less fortunate brothers is doubtful. The man of studious character and benevolent disposition will do more to elevate his profession than all the high chargers.

### A Great Clinic.

The London Dental Hospital, according to a New York newspaper, treated 100,000 patients last year, and made 35,000 operations. These figures seem incredible and it may be that the newspaper has misinterpreted them. A clinic of such magnitude is hardly possible in any dental colleges in this country. We can not quite understand how they could treat so many more patients than were operated upon. Thirty-five thousand operations for a college which teaches 200 days, which is the limit of any school in this country, would mean 175 operations each day. And it would mean, according to these figures, seeing 500 patients every day. The only inference is that no such operations or treatments are required in England as here. Five hundred patients would practically swamp the largest and best conducted clinic in America.

### The Value of Occlusion in Prosthodontia.

There is no part of dental practice that gives more cause for worry and complaint than the prosthetic department. It is such a difficult problem that many dentists absolutely refuse to do anything in this line, and turn their work over to an assistant or send it to the commercial laboratory. We have come to the conclusion that the most potent reason for this condition is due to the fact that no adequate consideration is given to the attainment of anything near natural occlusion of the teeth. Approximate occlusion is considered amply sufficient. Accurate and definite occlusion with proper adjustment of force direction is scarcely ever attained. The shape of the arches, the line of occlusion and the relations of the teeth to the process are not as carefully studied as they should be. It seems to be the rule to disfigure the mouth more often than to improve its appearance, even where there should be every inducement to attain artistic results. The close study now being given by the orthodontists to the subject of occlusion, if applied to prosthodontia, would do more to lift this branch out of its present chaotic condition than any other single thing. It would not only result in perfecting the occlusal lines, but would soon bring to bear on our tooth manufacturers a pressure that would result in their giving some attention to the matter of making artistic as well as practicable molds of teeth.

**Complimentary Banquet to Dr. J. L. Young.**

The Detroit Dental Society tendered to Dr. J. L. Young a banquet at the St. Claire Hotel in Detroit, April 29th, at which there were present nearly fifty of the prominent practitioners of Detroit and vicintiy. It was an occasion long to be remembered by all present, and also one that should impress its lesson upon the younger men in the profession. It wes essentially a tribute to the excellent skill and high character of one who, against adverse circumstances had built up in a large city, where he came unknown only a few years ago, a large and select practice, which he is now giving up to go to New York City to engage in the practice of Orthodontia as a specialty. It must require an optomistic mind to assume such an undertaking. Dr. Young, however, feels that this work is to be his future life work, and as be is a large man he feels willing to make the necessary sacrifice and cast his lot into the largest field in sight. His friends in Detroit and all who know him will follow his career with great interest, and confidently expect that he will carry the same ability and determination into his new sphere that has given him the enviable position he is leaving. The following resolutions were presented to him at the banquet. They were beautifully engrossed.

RESOLUTION.

*Whereas*, Dr. J. L. Young, for many years an ardent supporter of the highest ethical principles in the practice of his profession in our city, has decided to leave Detroit and locate in another field where he thinks he can be more useful, the Detroit Dental Society, representing the profession of its city, learns of his removal with great regret. In order that a suitable memorial of his professional life and activity may have some proper acknowledgement from his associates, we, as members of the Detroit Dental Society, desire to place on our records the following minutes:

*Resolved,* With great regret we learn of the departure of Dr. J. L. Young, from our city, where he has successfully established a practice on the highest ethical basis. His skill and devotion to professional interests have commended him to his associates, and his earnestness and honorable conduct have endeared him to all who have known and associated with him. We shall greatly miss his enthusiasm in our professional and social gatherings, and do most cordially commend him as an honorable and capable practitioner to any community or professional organization with which he may become associated, knowing that he will ever do his utmost to deserve the good will and confidence of all persons who may entrust their professional interests to him. Detroit, Mich., April 29, 1905.

